# Assessing the feasibility and appropriateness of verbal autopsy using contact information of the deceased from burial records in urban Bangladesh

**DOI:** 10.7189/jogh.16.04006

**Published:** 2026-01-16

**Authors:** Aniqa Tasnim Hossain, Ema Akter, Ridwana Maher Manna, Md Hafizur Rahman, Md Alamgir Hossain, Nasimul Ghani Usmani, Md Shahidul Islam, Tasnu Ara, Bibek Ahamed, Pradip Chandra, Abu Bakkar Siddique, SM Hasibul Islam, Mohammad Mamun-Ul-Hassan, Beth Tippett Barr, Tanvir Hossain AKM, Shafiqul Ameen, Anisuddin Ahmed, Md Toufiq Hassan Shawon, Shabnam Mostari, Mohammad Sohel Shomik, Qazi Sadeq-ur Rahman, Shams El Arifeen, Ahmed Ehsanur Rahman

**Affiliations:** 1 Maternal and Child Health Division, International Centre for Diarrhoeal Disease Research, Dhaka, Bangladesh; 2 Dhaka North City Corporation, Gulshan, Dhaka, Bangladesh; 3 Nyanja Health Research Institute, Salima, Malawi; 4 Management Information System, Directorate General of Health Services, Dhaka, Bangladesh; 5 Aspire to Innovate (a2i) Programme, Dhaka, Bangladesh

## Abstract

**Background:**

Bangladesh faces significant challenges in accurately documenting causes of death (COD), largely due to incomplete vital registration systems, which lack COD reporting. A substantial number of deaths occur outside health facilities, often without medical certification, leading to further gaps in mortality data. Verbal autopsy (VA) has emerged as a viable method in low-resource settings to bridge this gap. We aimed to explore the feasibility and appropriateness of using VA by tracking burial records’ contact information to enhance mortality documentation and inform health policies in the graveyards of urban Bangladesh.

**Methods:**

We employed an exploratory design using both quantitative and qualitative methods. We conducted VAs using the contact details from six graveyards’ burial records of Dhaka North City Corporation in Bangladesh, identifying participants through random sampling. In-depth interviews with data collectors, graveyard managers, and study participants provided insights into the feasibility and challenges of this process. We collected the data using the World Health Organization VA tool and assigned CODs using the InSilicoVA algorithm, applying thematic analysis to qualitative findings. We compared mortality trends with national data sets.

**Results:**

We conducted 531 VAs using the contact information from burial site records in Dhaka North City Corporation graveyards, with sub-optimal consent rates varying by location. The leading CODs were acute respiratory infections (21%) and cardiac disease (19%), demonstrating the practicality of obtaining COD from the VA, and the feasibility of collecting burial records and contact details, if consent rates could be improved. Qualitative findings indicated that using burial records for such data collection faces obstacles, including low response rates, socioeconomic disparities in participation, difficulty finding contacts, and sampling inconsistencies.

**Conclusions:**

We are the first to explore VA using contact information from burial records in urban Bangladesh. While the approach shows promise, the current feasibility results are of limited value without substantially improving consent coverage, representativeness, and standardisation. Only with these improvements can this method meaningfully strengthen COD documentation and provide reliable insights into population-level mortality trends.

Effective vital registration systems are either incomplete or unavailable in most low- and middle-income countries, making it difficult to accurately provide information on deaths [1–4]. Civil registration refers to the systematic, comprehensive, enduring, and mandatory documentation of vital events within a nation's population, in accordance with its legal framework [5]. The United Nations has identified ten key events to be recorded, including live birth, death, foetal death, marriage, divorce, marriage annulment, judicial separation, adoption, legitimation, and recognition [6]. Vital statistics encompass the compilation of data on essential life events, including details about the events themselves and the individuals involved [7]. These statistics are primarily derived from the civil registration system, although additional sources such as population surveys and censuses also contribute to the collection of vital demographic data. Understanding the causes of mortality is crucial for improving health outcomes and reducing deaths [5,6]. This information is necessary for redesigning the existing policies and strategies to avert preventable deaths [7].

Documentation of vital events, such as births and deaths, often lacks reliable cause of death (COD) information. There is a significant gap in evidence regarding COD, presenting challenges to accurately identifying the nation's health burden and addressing critical areas of concern in Bangladesh [1,8]. The Bangladesh Bureau of Statistics (BBS) conducts a surveillance with a nationally representative sample to monitor key population and demographic indicators [9]. However, information regarding the COD is not systematically collected by ensuring a global standard [10,11]. A substantial portion of deaths in Bangladesh occur outside health facilities, leaving them undocumented and without medical certification of COD [12]. Although the World Health Organization (WHO) recommends the use of this form for the facility death to ensure standardised reporting, most deaths in Bangladesh are not medically certified [12,13]. Of those that are, COD reporting is not standardised and is very rarely documented using the International Classification of Diseases (ICD) system [14].

In countries such as Bangladesh, where formal COD documentation systems are weak or incomplete, verbal autopsy (VA) emerges as an alternative tool for determining individual COD and estimating cause-specific mortality fractions in the absence of a complete vital registration system [3,15]. The process of VA involves gathering information on the circumstances and symptoms preceding death through structured interviews with individuals familiar with the deceased, such as family members or caregivers [16]. This method has been widely adopted in low-resource settings to bridge gaps in mortality data and provide insights into COD distributions. While VA has been widely used, its feasibility and acceptance using deceased people’s contact details in burial records from graveyards remains unexplored. Burial records in graveyard registers typically include details such as the deceased person's name, date of burial, plot location, and family contact information. These records can be used to reach out to family members for VAs, using the provided contact details or to submit inquiries to the graveyard authorities. These burial records may offer advantages over household surveys by capturing deaths more continuously and enabling potential linkage with municipal registers, thereby providing a complementary data source for mortality surveillance.

We seek to address this gap by investigating the feasibility and appropriateness of conducting VAs for deceased individuals buried in selected urban graveyards, where documentation on COD is minimal or non-existent. We further seek to assess the distribution of COD through VA in these settings, offering an innovative approach to understanding mortality patterns in populations typically excluded from formal health data systems. By leveraging cemetery data records and implementing VA methods, we aim to create a foundation for generating actionable evidence in environments with limited mortality data, thereby avoiding repetition of COD documentation. We expect the findings of this study to provide important insights into the practicality of using graveyard records as a basis for the VA and their potential to improve COD data. Additionally, the findings will highlight gaps in existing mortality documentation systems and offer recommendations for integrating VA into broader public health and civil registration strategies.

## METHODS

### Study design

We conducted an exploratory study using both quantitative and qualitative methods. We assessed feasibility through completion rates and logistical practicality, and appropriateness through participant receptivity, ethical acceptability, and data quality.

We contacted 1500 families based on burial site records and received 531 verbal consents. The recorded deaths occurred between October 2022 and December 2023, with VA serving as a tool to gather insights where formal documentation of COD was minimal.

We qualitatively explored the feasibility of conducting VAs for individuals buried in selected city corporation graveyards. We conducted nine in-person in-depth interviews with participants from diverse backgrounds: two graveyard managers (Mohorar), two VA data collectors, and five VA study participants.

Through interviews with graveyard managers, we aimed to understand their role in VA conduction, as they provided death information to the VA team and addressed concerns raised by study participants before the autopsy process. In conversations with VA participants, we focused on their experiences and perceptions when engaging with data collectors.

We selected the sample for VA using simple random sampling. The sampling frame comprised 1500 recorded burials across six graveyards, from which we drew random samples using a computer-generated list. We excluded duplicate and incomplete records before selection. Field staff and graveyard managers tracked the deceased's medical certificates and documented burial events. To minimise participants’ distress and maximise recall accuracy, data collectors contacted families after a gap period of approximately two weeks after the death of the buried person in the graveyard. Our team of VA data collectors contacted family members to schedule visits to understand the true COD using the detailed WHO VA tool.

### Study site

We carried out this study on the deaths buried in six Dhaka North City Corporation graveyards (Table S1 in the **Online Supplementary Document**). Information on buried individuals was documented on the website, Digital Graveyard System [17], a web-based platform that contains detailed information about each deceased person. This system enabled interviewers to identify the deceased and contact their families. By consolidating this information online, the platform facilitated easier and more efficient communication with family members. In addition, the website presents data from each of the six graveyards separately, allowing users to access relevant information quickly without the need to visit each site individually. We conducted the interviews at locations convenient to or suggested by the respondents (Appendix S1 in the **Online Supplementary Document**).

### Study population

The study population comprised graveyard managers and family members of deceased individuals buried in those selected graveyards. Graveyard managers played a crucial role in providing burial records and facilitating the VA process, as they were responsible for maintaining site records and interacting with bereaved families. We engaged family members of the deceased in VA interviews to reconstruct the circumstances surrounding the deaths.

### Data collection methods

We collected data using structured interviews through a VA questionnaire. Initially, we contacted the participants by phone using the contact details from the burial records in the graveyards. After that, we conducted an in-person interview if consent was received for VA. Additionally, we conducted qualitative interviews with VA data collectors, graveyard managers, and family members of deceased individuals to gain deeper insights into the feasibility and appropriateness of VA in burial sites.

We removed all identifying information during transcription and analysis to ensure anonymity. We obtained informed consent prior to participation, as approved by the institutional review board, and informed participants that they could withdraw at any time. Data collectors were trained to handle sensitive topics with empathy and to minimise distress, particularly in cases involving suicide or domestic violence.

### Data collection tool

For VAs, we used the WHO standard VA tool, which was updated for the context of the COVID-19 pandemic in 2020 [18]. The tool was translated into Bangla based on the Bangladesh Demographic and Health Survey (BDHS) 2022 VA questionnaire [19].

For the in-depth interviews, we used an interview guideline to explore the feasibility and appropriateness of conducting VA in graveyards, and to assess perceptions, challenges, and implementation strategies. Interviews examined participants' views on VA data collection in burial sites, barriers to providing accurate death information, and the most effective approaches for gathering burial records. Discussions also covered field challenges, potential mitigation strategies, concerns regarding VA methodology, and differences between graveyard-based and household-based VA data collection. Additionally, the tools explored the benefits of conducting VAs in graveyards, emphasising their role in strengthening mortality surveillance and improving documentation of deaths where formal medical records are limited (Appendix S2 in the **Online Supplementary Document**).

### Data analysis

#### Quantitative analysis

For the quantitative analysis, we calculated the proportions of cause-specific mortality across age groups (neonates, <5 years, and ≥12 years) and reported 95% confidence intervals for each cause. To determine the COD, we used the *R* package InSilicoVA [20], which estimates the most likely joint probability distribution of cause-specific mortality fractions and probabilities of each cause for each death. InSilicoVA is open-source and available for the *R* programming language [18].

Additionally, we used secondary data from the Sample Vital Registration System (SVRS) 2023 and BDHS 2022 to compare findings. The SVRS, operated by the BBS, is a nationally representative system that collects vital events data, including COD, primarily through community-based reporting. However, SVRS often relies on a single reported symptom or summary description from family members, without standardised medical certification. In our analysis, we used the COD distribution from SVRS 2023 to compare with our VA findings from the burial records [9].

The BDHS collects COD data through VA interviews with family members or caregivers of the deceased. These interviews help determine the probable COD, which is then classified by a panel of physicians using the ICD-10 coding system. The BDHS employs a two-stage stratified cluster sampling design to ensure national representativeness, and its findings contribute to understanding mortality trends and health interventions in Bangladesh [19]. It is important to note that we compared our results with SVRS and BDHS data as illustrative rather than formal validation exercises, given differences in their respective methodologies.

### Qualitative analysis

For the qualitative component, we transcribed audio-recorded interviews *verbatim* in Bangla. We reviewed the transcripts multiple times to ensure familiarity with the content, and then systematically coded them using an inductive approach. We purposively selected graveyard managers, data collectors, and bereaved families to capture diverse perspectives. Two researchers (MAH and MSI) conducted inductive coding, with regular discussions to resolve discrepancies. We developed a coding framework based on emerging concepts identified during the review and coding, which was then organised into a matrix of broader themes and sub-themes. We conducted a thematic analysis to identify patterns across different participant groups, including graveyard managers, data collectors, and family members, to gain a deeper understanding of their perspectives. We selected representative quotations to illustrate key findings and to support the interpretation of themes. We synthesised the findings under six thematic domains: perceived importance of VA in mortality data collection, barriers to participation, challenges in identifying respondents, comparisons with household-based VA, socioeconomic influences, and ethical concerns. We manually carried out the analysis to ensure reliability and consistency in interpretation.

## RESULTS

Out of 1500 families contacted using burial site records, we conducted 531 VAs after obtaining consent. The proportion of consent received varied across the selected graveyard sites, reflecting differences in community response and accessibility (**Table 1**).

**Table 1:** VA consent and completion across selected graveyards

	Families contacted (n = 1500)	Received consents*	Conducted VAs*
**Uttara 4 graveyard**	127	49 (38.6)	49 (100.0)
**Uttara 12 graveyard**	120	48 (40)	48 (100.0)
**Uttara 14 graveyard**	33	15 (45.5)	15 (100.0)
**Mirpur Buddhijibi (Martyred intellectual graveyard)**	513	171 (33.3)	171 (100.0)
**Rayerbazar graveyard**	707	248 (35.1)	248 (100.0)
**Banani graveyard**	0	0	0

VA – verbal autopsy

*Values presented as n (%).

Among the graveyards, Uttara 14 graveyard had the highest consent rate (45.5%), followed by Uttara 12 graveyard (40%) and Uttara 4 graveyard (38.6%). Rayerbazar graveyard had a 35.1% consent rate, while Mirpur Buddhijibi (Martyred intellectual graveyard) had the lowest at 33.3% among locations with successfully conducted VA. No VAs were conducted in Banani, as no families were contacted or provided consent.

**Figure 1:**
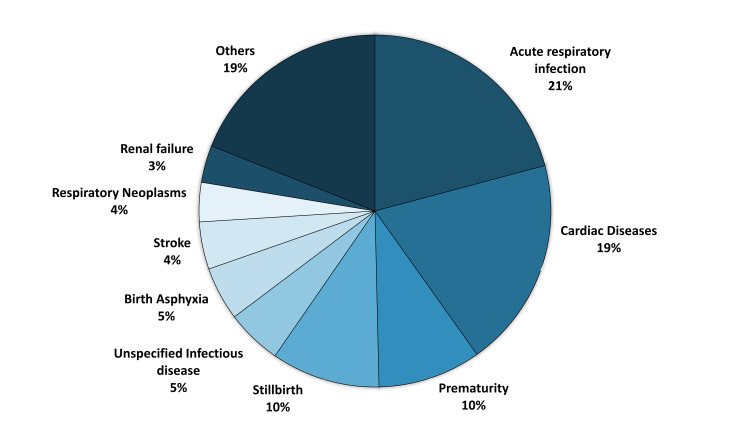
Percent distribution of COD from VA using information from the burial site (n = 527). COD – cause of death, VA – verbal autopsy.

Acute respiratory infections, including pneumonia, were the most common COD, accounting for 21% of cases, followed closely by cardiac disease (19%). Prematurity (10%) and stillbirth (10%) were significant contributors, indicating a notable burden of neonatal mortality (**Figure 1**). Unspecified infectious diseases (5%) and birth asphyxia (5%) were additional causes, particularly affecting infants. Stroke (4%), respiratory neoplasms (4%), and renal failure (3%) contributed to deaths, primarily among older individuals. The ‘Others’ category (19%) represents the combined total of multiple individual causes, each contributing <1% to the overall mortality. Although these causes are specifically identified, we grouped them for summary purposes because of their low individual frequencies.

Among neonates, the leading CODs were prematurity (35%), macerated stillbirth (30%), and birth asphyxia (18%), followed by neonatal pneumonia (9%) and fresh stillbirth (8%) (**Table 2**). Post-neonatal deaths mostly occur due to acute respiratory infections (70%). Overall, in the under-five group, prematurity (28%) remained the most common cause, with macerated stillbirth (24%), acute respiratory infections including pneumonia (14%), and birth asphyxia (14%) contributing significantly. Among individuals aged ≥12 years, the predominant CODs were acute respiratory infections (21%), acute cardiac disease (24%), unspecified infectious disease (9%), respiratory neoplasms (7%), and stroke (7%).

**Table 2:** COD across three age groups

	n (%)
**Neonate**	143
Prematurity	50 (35)
Macerated stillbirth	43 (30)
Birth asphyxia	26 (18)
Neonatal pneumonia	13 (9)
Fresh stillbirth	11 (8)
**<5 years**	180
Prematurity	50 (28)
Macerated stillbirth	43 (24)
Acute respiratory infections	25 (14)
Birth asphyxia	25 (14)
Neonatal pneumonia	13 (7)
**≥12 years**	336
Cardiac disease	81 (24)
Acute respiratory infection	71 (21)
Unspecified infectious disease	30 (9)
Stroke	24 (7)
Respiratory neoplasms	24 (7)

**Figure 2:**
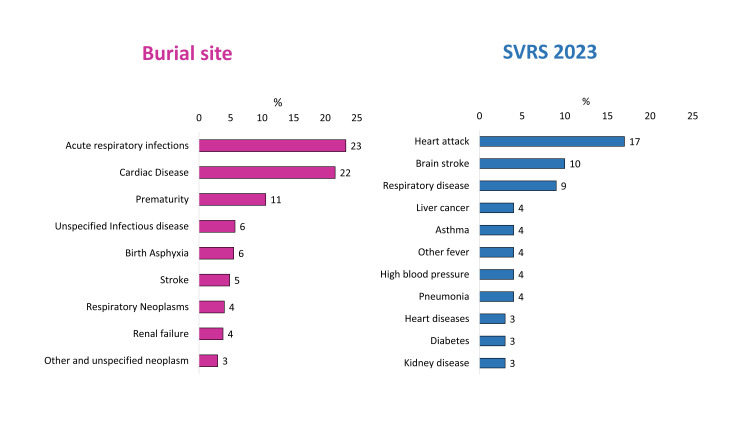
Comparison of major CODs between burial site records (n = 473) and the SVRS. **Panel A.** Burial site. **Panel B.** SVRS 2023. COD – cause of death, SVRS – Sample Vital Registration System.

In the burial site records, the leading CODs were acute respiratory infections (23%) and cardiac disease (22%), reflecting a high prevalence of respiratory and cardiovascular conditions (**Figure 2**, Panel A). Prematurity (11%) was also a significant contributor, indicating concerns about neonatal mortality. Other notable causes included unspecified infectious diseases (6%), birth asphyxia (6%), stroke (5%), respiratory neoplasms (4%), and renal failure (4%), which primarily affect different age groups.

By contrast, the SVRS 2023 data identified heart attack (17%) as the most prevalent COD, followed by brain stroke (10%) and respiratory diseases (9%), emphasising non-communicable disease burden (**Figure 2**, Panel B). Unlike the burial site records, the SVRS recorded cases of liver cancer (4%), asthma (4%), other fever-related deaths (4%), high blood pressure (4%), and pneumonia (4%), indicating a broader spectrum of chronic and acute conditions.

**Figure 3:**
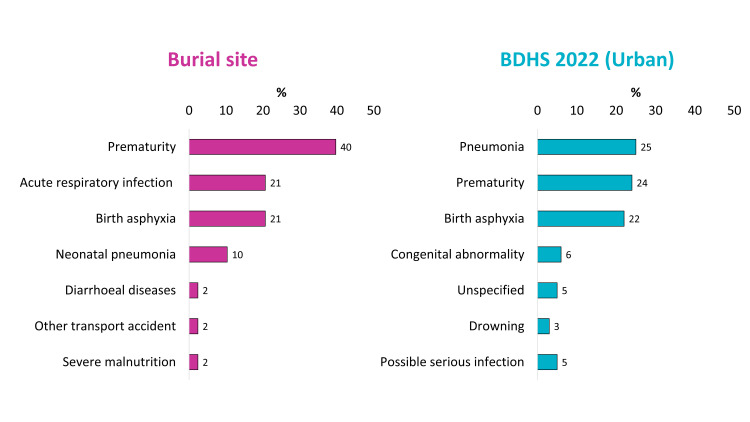
Comparison between major COD estimates between burial site records and urban data from BDHS 2022. **Panel A.** Burial site. **Panel B.** BDHS 2022 (Urban). COD – cause of death, BDHS – Bangladesh Demographic and Health Survey.

In burial site data, prematurity was the most prevalent COD (40%), whereas in BDHS 2022 urban data, pneumonia ranked highest (25%) (**Figure 3**, Panels A and B). Both sources reported prematurity as a major contributor, though with differing proportions (40% *vs.* 24%). Birth asphyxia showed similar prevalence across both data sets (21% in the burial site *vs.* 22% in BDHS 2022), indicating consistency in neonatal mortality trends.

Acute respiratory infection was reported at 21% in the burial site data, but was not explicitly listed in BDHS 2022. Instead, pneumonia (25%) was reported, suggesting a possible classification overlap. Neonatal pneumonia was significantly present in burial site records (10%), whereas congenital abnormality (6%) was reported in BDHS 2022, showing variance in neonatal conditions recorded.

Lower-frequency causes varied between sources. Diarrhoeal diseases (2%), other transport accidents (2%), and severe malnutrition (2%) were reported in burial site data, whereas BDHS 2022 urban data included drowning (3%), unspecified causes (5%), and possible severe infection (5%).

### Perceived importance of VA in mortality data collection

#### Importance of VA in mortality data collection

The deceased's family members regarded the VA as a vital tool, as it provides an opportunity to maintain accurate data sets on the COD. This information can assist governments and research institutions in understanding mortality trends, including the number of people who died and whether unclear or suspicious circumstances surrounding a death were involved. One data collector noted:

This process is important because it helps bring the real story or truth to light, even if the person has passed away, at least we know what really happened to the person. *– (IDI, VA data collector, male, 32 years)*

### Barriers to participation

#### Role of graveyard managers in VA data collection

Graveyard managers perceived that VA data could be collected efficiently directly from the graveyard death registers, as these often include COD information, thereby minimising the need for household visits. They have highlighted both the challenges and the importance of implementing VA processes while addressing participants' concerns and utilising existing data efficiently. One graveyard manager stated:

I believe verbal autopsy data can be efficiently collected using burial site records, contact details. While there are challenges in implementing this process, I recognise its importance in streamlining data collection, addressing participant concerns, and effectively utilising existing records. *– (KII, graveyard manager, male, 36 years)*

### Challenges in discussing COD during VA interviews

During initial communication with data collectors before VA data collection, participants often expressed discomfort when asked about the COD, especially for sensitive issues such as suicide. Despite explanations about the role of data collectors and the purpose of the VA process, some participants feared potential legal or police involvement.

This concern was particularly pronounced in cases where sensitive family dynamics were involved, as exemplified by one participant’s account:

My sister died by suicide after experiencing physical violence from her husband. When an unknown person calls, it raises concerns about their identity and purpose, are they from law enforcement? *– (IDI, VA data collector, female, 28 years)*

Such apprehensions hindered their willingness to engage. Nevertheless, family members acknowledged the value of VA in clarifying the true COD.

### Challenges in identifying respondents

#### Challenge in identifying the appropriate respondent by phone

Using burial site records, VA faces significant challenges, particularly regarding the registered mobile numbers of deceased individuals. These numbers are often linked to male family members or non-household individuals, rather than the intended participant, who may possess detailed knowledge of the circumstances surrounding the death. As a result, it becomes difficult for data collectors to obtain accurate and comprehensive information required for determining the COD. This disconnect can lead to incomplete or less reliable data, as those contacted may lack first-hand experience or the necessary context to provide detailed responses. As one data collector explained:

Most of the phone numbers were not helpful, often, it was the husband’s number or someone who didn’t even live in the same house. We had to keep calling different people to find someone who actually knew what happened. *– (IDI, VA data collector, male, 32 years)*

Addressing this issue is critical for enhancing the effectiveness of VA data collection based on burial site records, ensuring accurate mortality documentation and meaningful health insights.

#### Low response rates

Graveyard-based VA refers to a method of identifying deaths through burial site records and subsequently contacting family members, often via phone, for follow-up interviews rather than through a household listing. This approach is beneficial in settings where deaths are not routinely tracked through household visits or civil registration systems.

Contacting participants for graveyard-based VAs presents significant challenges, with a notably low response rate. A data collector described:

During the verbal autopsy data collection, I used to call on the phone, but most of the time they did not pick up. If you call with 100 phone numbers, very few people would agree, and the rest would not respond. It has also happened that someone gave a date and then didn’t show up. However, there was no such problem in household-based verbal autopsy which are conducted as a part of household. *– (IDI, VA data collector, male, 32 years)*

### Comparisons with household-based VA

#### Comparison of VA based on household surveys and using burial record contacts

Typically, VA is implemented as part of a household-level survey or surveillance system. In household-based approaches, VAs are scheduled after identifying deaths through a prior survey or surveillance system through known addresses and death lists. This approach allows data collectors to engage directly with family members in private, familiar settings, where they can gather detailed information.

In contrast, when VAs are conducted using contact details obtained from graveyards, the information is often limited. This approach relies heavily on reaching participants by phone and arranging interviews in public spaces, such as parks or tea stalls, due to the absence of formal household connections. These public settings can pose challenges related to data quality, confidentiality, and participant comfort. A data collector stated:

Household visits allowed us to sit with family members and gain a full understanding of events. In contrast, interviews based on graveyard-records contact details often took place in public settings like tea stalls, where asking sensitive questions was challenging, and participants sometimes felt uneasy speaking openly. *– (IDI, VA data collector, female, 28 years)*

#### Lack of generalisability

Sampling in graveyard-based VA studies faces distinct challenges due to the diverse geographic origins of individuals buried at these sites. Unlike household-level VAs, which utilise systematic sampling to ensure a structured selection of participants within clearly defined communities, graveyard-based sampling often lacks this consistency. Burial sites accommodate individuals from various locations, making it difficult to implement standard sampling methods and potentially leading to representation bias. One graveyard manager explained:

People come from many different places to bury here. Sometimes we don’t even know the full address, just a name and phone number, and they might even be from another district. *– (KII, graveyard manager, male, 42 years)*

### Socioeconomic influences

#### Impact of socioeconomic status on VA participation

High refusal rates from wealthier individuals and the inability to capture all deaths, particularly those buried in private or community graveyards, compound the challenges associated with graveyard-based VA data collection. A data collector conveyed:

Individuals from higher social classes were not interested in providing death-related information. They often became visibly upset, repeatedly asked various questions, and showed no interest in providing verbal autopsy data. *– (IDI, VA data collector, female, 28 years)*

### Ethical concerns

#### Privacy concerns and resistance

During burials, family members provide personal information about the deceased, including name, age, sex, national identification details, and contact information. While some relatives view the use of this data for VA as beneficial in determining the true COD, others express frustration and discomfort upon learning that death information is sourced from graveyards. Concerns arise over privacy breaches, unexpected calls from unknown individuals requesting VA data, and discomfort when enumerators visit households based on graveyard records. These factors contribute to varying levels of acceptance and resistance toward graveyard-based VA data collection. A participant noted:

I find it unsettling when unknown callers request death information gathered from graveyards. Such details are not meant for public access. When someone passes away, we should honour their memory and good deeds rather than dwell on the cause of death, as only the Almighty knows the truth! *– (IDI, male participant, 40 years)*

## DISCUSSION

To our knowledge, we are the first to explore the feasibility of identifying COD undertaking in VA using deceased people’s contact information from burial records at urban graveyards in Bangladesh. Bangladesh lacks a well-established system for obtaining reliable COD statistics, which would provide trustworthy information on the overall national health status. To address this gap, the feasibility of conducting VA using the contact information from burial records at a later period is promising, but certain limitations must be considered. While this data could serve as a valuable source for determining COD, careful interpretation is required to avoid misclassification or incomplete conclusions. Privacy concerns, participant hesitancy, and emotional resistance, especially in sensitive cases like suicide or domestic violence, should also be addressed. The current coverage of graveyards where VAs were done using the contacts from burial records is limited, which can affect the accuracy and representativeness of mortality trends [21]. Therefore, unless future implementation strategies achieve significantly higher consent rates, the broader utility of this approach for population-level mortality surveillance will remain limited. Scaling up the use of burial site records for VA requires systematic improvements in coverage and the integration of data with other mortality surveillance systems for validity assessments. If burial registers were carefully revised, the burial records' contact details could make VA a practical and effective tool for strengthening COD documentation.

We observed varied and low consent rates among those contacted for VA. The differences in consent rates across graveyards suggest varying levels of willingness among families to participate in VA, potentially influenced by factors such as awareness, acceptance, and logistical feasibility at different sites. Only one-third of the cases we contacted over the phone consented to conducting such interviews in person. In the Banani graveyard, which generally belongs to the higher socioeconomic class in Dhaka, no permission was granted to access the burial records for further use by the VA. Therefore, the consent rates are strongly associated with social status and could introduce selection bias in the COD distribution, as the data will systematically exclude people from higher socioeconomic classes. This finding aligns with existing research on the challenges of VA implementation, notably regarding consent rates and socioeconomic disparities. Studies have shown that VA acceptance varies across different social and cultural contexts, with higher socioeconomic groups often exhibiting greater reluctance to participate due to concerns over privacy, legal implications, and perceived intrusion [22]. In wealthier communities, concerns about confidentiality and potential misuse of data may contribute to lower consent rates, as observed in Banani graveyard. This aligns with findings from the WHO, which emphasises that VA implementation must consider cultural and socioeconomic factors to ensure representative mortality data collection [23].

While we explored the use of burial records to conduct VAs, several challenges emerged that limit broader feasibility. With this method, we were able to capture a broad spectrum of mortality causes, particularly those related to neonatal deaths, respiratory infections, and cardiovascular diseases. Our findings indicate that acute respiratory infections (21%) and cardiac disease (19%) were the leading COD, aligning with global mortality trends where respiratory and cardiovascular conditions remain dominant contributors to mortality [24,25]. However, the low consent rate significantly affected participation and introduced notable selection bias, especially in cases involving sensitive causes. Despite its feasibility, coverage limitations remain a concern. Scientifically, data derived from burial records may lack generalisability due to the selective nature of such records [26]. Not all deaths are registered at public cemeteries of the specific region where we worked; individuals may be buried in private, community, or family graveyards, particularly in rural or remote areas [27]. This introduces a sampling bias, as the data disproportionately represent urban or specific demographic groups [28]. The WHO VA standards emphasise the need for comprehensive data collection to ensure representative mortality estimates [23].

Furthermore, locating the appropriate participant to provide VA data via phone contacts was challenging. The burial site data often contains incomplete or inaccurate contact information, which raises questions about informed consent [29]. Also, the registered mobile numbers linked to burial records are often associated with male relatives or non-household members, as evidenced in qualitative research on VA implementation. This limits data collectors' ability to access participants with firsthand knowledge of the circumstances surrounding the death, reducing the reliability of the information. Therefore, expanding burial site-based VA could enhance the utility of COD information for policy decisions. Still, methodological refinements, such as improving participant recall and reporting accuracy, ensuring systematic data collection, and ensuring representativeness from all geographical and socioeconomic groups, are necessary to strengthen its reliability.

The comparison of mortality patterns between burial site records, SVRS 2023 [9], and BDHS 2022 [19] urban data demonstrated significant variations in reported COD, reflecting differences in data collection methodologies and classification criteria. The COD using VA following burial site records emphasise acute respiratory infections (23%) and cardiac disease (22%) as leading causes. In contrast, SVRS 2023 data prioritise heart attack (17%) and brain stroke (10%), suggesting a greater burden of non-communicable than infectious disease mortality. Similarly, BDHS 2022 urban data report pneumonia (25%) as the leading COD, in contrast to burial site records, where prematurity (40%) is most prevalent. It is important to note that all national data sources also have their own limitations. The SVRS of the BBS serves as the primary source for reporting the national COD for the entire population in Bangladesh. However, several gaps and limitations affect the accuracy of its data. One major concern is the method used to determine the COD, as SVRS relies on a single-question response from family members. COD assignments in the SVRS were not based on ICD codes, which limited standardisation and reduced comparability with our findings and other national or international data sets. Additionally, local registrars frequently lack adequate training in COD classification, leading to inconsistent reporting. The absence of standardised classification systems, such as the ICD, further hinders comparability with international studies and benchmarks. Another limitation arises from the selection of participants, as any family member may provide information, regardless of their knowledge of the circumstances surrounding the death. This can result in misclassification, affecting overall mortality assessments [30]. Moreover, SVRS does not collect data on disease symptoms, preventing a comprehensive determination of immediate, underlying, or contributory COD, as required by global standards [9]. Finally, the COD assignment and data collection are not conducted independently, which directly compromises data quality. To improve the accuracy of SVRS, standardised training for registrars, enhanced participant selection, and integration with ICD coding systems are crucial. Also, the BDHS has provided only the representative COD for children under five. However, the survey is currently facing sustainability concerns due to global politics. In the absence of a reliable national source of COD data, conducting VA could act as a complementary data source for policy decisions with certain improvements.

It is important to note that conducting VAs using burial records introduces both ethical considerations and challenges related to generalisability, which limit the robustness and applicability of the collected data. From an ethical standpoint, the privacy of the deceased and their family must be respected, particularly when sensitive personal details are shared. This is especially crucial in contexts where the COD, such as suicide, violence, or infectious diseases, may carry social stigma or legal implications. Studies have noted the emotional distress experienced by both participants and interviewers during VA data collection, particularly when discussing traumatic deaths [31]. Additionally, the lack of a standardised protocol to ensure the anonymity of data collected from burial records may lead to breaches of confidentiality, contributing to mistrust among participants. This underscores the need for ethical safeguards, such as obtaining informed consent, ensuring confidentiality, and conducting interviews at appropriate times to minimise distress, which remained a challenge when collecting burial records from graveyards to conduct VAs.

This implementation study on the feasibility and appropriateness of conducting VA using burial site records of urban Bangladesh offers both strengths and limitations. A key strength is our comprehensive approach, integrating quantitative mortality data from burial records with qualitative insights from graveyard managers, data collectors, and families, providing a holistic assessment of VA feasibility. As the first study of its kind in Bangladesh, it fills an important research gap and presents practical policy implications for improving COD documentation. Additionally, we compared burial-site VA findings with other national COD data to assess data reliability and cross-validate mortality trends. However, limitations remain, including selection bias, as higher socioeconomic groups were less likely to grant consent. Coverage gaps also exist, as not all deaths are registered at public cemeteries, particularly in urban areas where private and community graveyards are used. While the completeness of death registration in public cemeteries in Bangladesh is not well documented, it is likely partial, and no formal estimate currently exists to quantify this gap. Therefore, selection bias was evident because we excluded private and community graveyards, which wealthier families often use. This systematically underrepresents deaths among higher socioeconomic groups. Non-response bias was also likely, since families declining participation may have differed systematically from participants (*e.g.* deaths from sensitive causes such as suicide). These limitations reduce the generalisability of our findings. Future work should explore strategies to improve consent rates and engage private/community graveyards to ensure more representative data. Furthermore, data accuracy challenges arise from inappropriate participant selection and interview location, unlike standard VAs following household surveys. One key limitation of this study is the lack of sufficiently reliable and comparable data for all COD in Bangladesh. Existing mortality survey and surveillance systems have significant gaps, including incomplete COD classification, reliance on participant-reported information, and absence of standardised coding systems such as the ICD [19,32]. Another limitation is that harmonisation of COD categories across SVRS, BDHS, and our study was not feasible, as we used COD data directly from the published reports. Thus, observed differences may partly reflect classification artefacts rather than true epidemiological variation. These inconsistencies limit direct comparisons across data sets, making it challenging to validate and cross-reference burial-site-based VA findings with national estimates. Moreover, InSilicoVA is designed for use with VA data from community deaths, where medical certification is typically unavailable. Direct validation against medically certified COD is therefore limited. However, future studies should compare InSilicoVA-derived causes with physician review-based COD results to assess accuracy and misclassification, especially for clinically complex conditions, such as differentiating stroke from cardiac disease. Also, our qualitative analysis is limited by the absence of formal intercoder reliability statistics, which may have affected coding consistency. Lastly, while we took measures to protect confidentiality, we recognise that discussing sensitive deaths (*e.g.* suicides) caused distress for some families. Counselling or referral mechanisms were not in place, which we acknowledge as a limitation. Future studies should integrate psychosocial support protocols in line with WHO VA ethics guidance.

### Policy implications

To enhance the feasibility and effectiveness of the burial record-based VA, policymakers could focus on several key areas. Expanding coverage to include private and community burial sites would help ensure more representative mortality data, capturing a broader range of demographic groups. This would require overcoming significant logistical and legal barriers, including the absence of centralised registers and data privacy constraints. To strengthen mortality surveillance, we propose the digitalisation of burial records, training for cemetery staff in systematic record-keeping, and the integration of burial registers into Civil Registration and Vital Statistics systems. This should be supported by the development of a clear legal framework for data access and complemented by pilot testing, active stakeholder consultation, and cost-effectiveness studies before any wider implementation. Ethical safeguards must also be strengthened, including clear informed consent procedures and robust privacy protections for participants, to maintain trust and adherence to ethical research standards. Additionally, targeted awareness campaigns should be developed to increase participation among higher socioeconomic groups, who often exhibit reluctance due to privacy concerns or perceived intrusion. By addressing these areas, burial record-based VA can become a more reliable and effective tool for improving mortality surveillance and informing health policy decisions.

## CONCLUSIONS

To scale up the use of deceased people’s contact information from burial site records for the VA requires systematic improvements, such as ensuring reliable registration practices and standardising protocol for VA data collection. While our study demonstrated feasibility, low consent rates and socioeconomic bias limit broader applicability. Burial record tracking-based VAs should not yet be considered a routine surveillance tool without substantial efforts to improve acceptability and coverage. If appropriately expanded and refined, VAs at a later period, based on burial records contact details, could become a practical and effective tool for strengthening COD documentation in settings with weak civil registration systems.

## Additional material


Online Supplementary Document
