# Supplementary material for: Assessing the feasibility and appropriateness of verbal autopsy using contact information of the deceased from burial records in urban Bangladesh
Source: J Glob Health. 2026 Jan 16;16:04006. doi: 10.7189/jogh.16.04006 (PMC12810587; doi:10.7189/jogh.16.04006)
Supplement: Online Supplementary Document [file jogh-16-04006-s001.pdf]

Supplement to: Hossain AT, Akter E, Manna RM, Rahman MH, Hossain MA, Usmani NG, Islam MS, Ara T, Ahamed B, Chandra P, Siddique AB, Islam SMH, Mamun-Ul-Hassan M, Barr BT, Hossain AKMT, Ameen S, Ahmed A, Shawon MTH, Mostari S, Shomik MS, Rahman QS, Arifeen SE, Rahman AE. Assessing the feasibility and appropriateness of verbal autopsy using contact information of the deceased from burial records in urban Bangladesh. J Glob Health. 2026;16:04006.

Table S1. List of graveyards under DNCC

SL. no	Religion	Graveyard Name	Area (m^2)	Address	GPS Location		Records available since
					Latitude	Longitude	
1	Muslim	Uttara Sector-14 Cemetery	8406	Road-21 / A,51 no Ward, Sector-14,Uttara	23.863377	90.385388	2019
2	Muslim	Uttara Sector-04 Graveyard	8037	Road-02,Uttara Sector-04	23.862757	90.405081	1995
3	Muslim	Shahid Buddhijibi Graveyard (Martyred Intellectual Graveyard)	55249	Mirpur 1 Mazar Road, Mirpur-1,Dhaka-1216	23.793398	90.345976	1995
4	Muslim	Banani Graveyard	39283	Road-27,Banani	23.802477	90.405269	1974
5	Muslim	Rayer Bazar Graveyard	21505	Sadarghat-Gabtoli Road,Basila,Mohammadhpur	23.748926	90.358893	2016
6	Muslim	Uttara 12 No Sector Graveyard	28324	Uttara 12 No. Sector	23.871474	90.379337	2012

Appendix S1

The snapshot illustrates the home, graveyards, way to bury a grave, policy and other services of Sector 4 Uttara Graveyard. Similar page appears for the other graveyards.

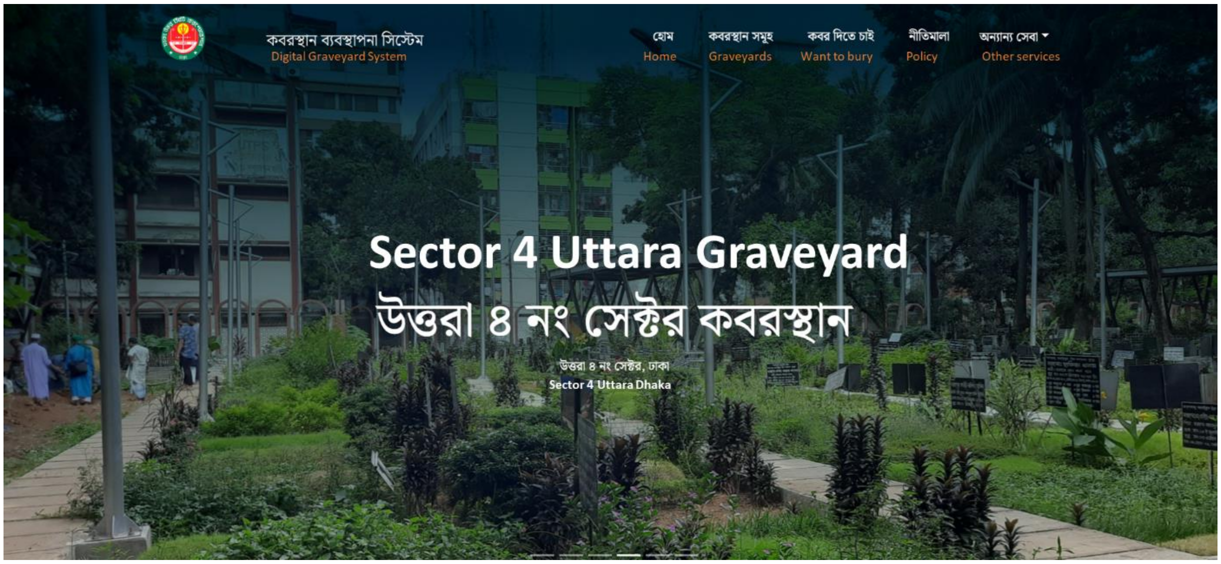

Figure S1. Snapshot of a graveyard web page.

The snapshot displays six graveyards, highlighting space availability and the number of individuals buried.

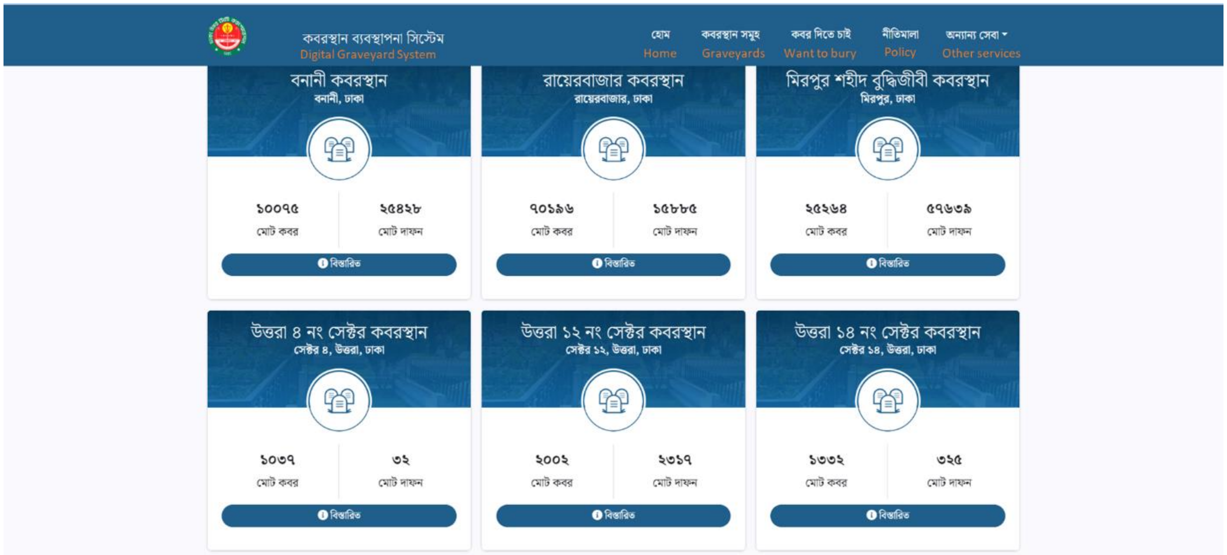

Figure S2. Snapshot of the six-graveyard web page.

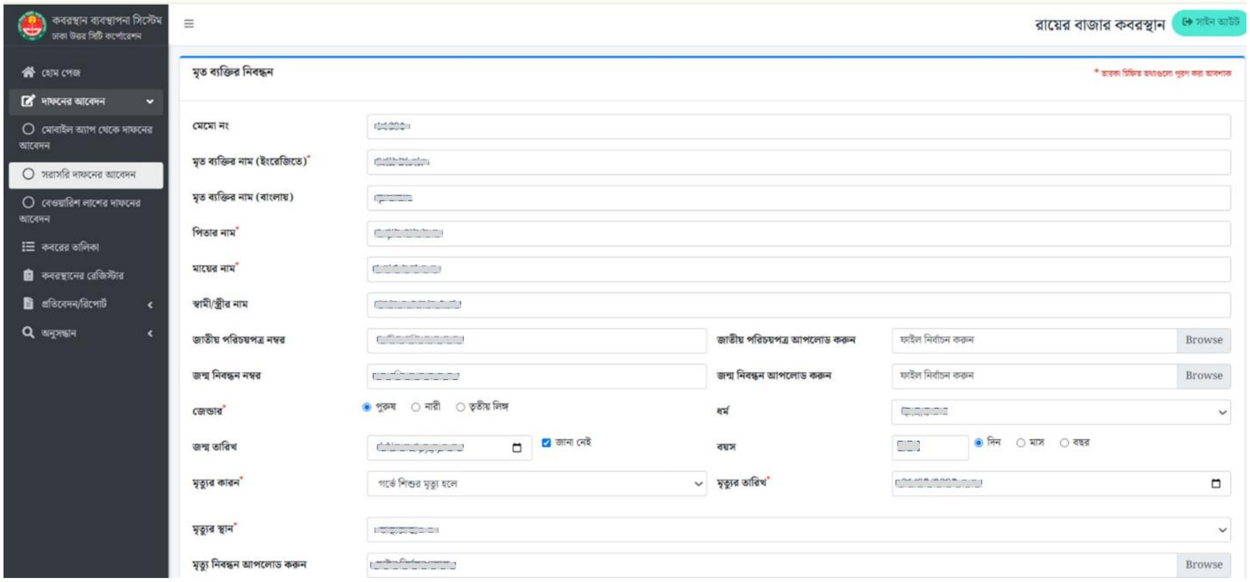

Figure S3. Biographical information of the deceased individual

Appendix S2

Qualitative Guideline for Conducting Key Informant Interviews with Graveyard Managers

General Information

- Respondent's age, marital status, education, occupation, and duration of work and responsibility at the burial site.

Verbal Autopsy-Related Information

- How do you perceive the necessity of cause-of-death (COD) data from burial sites? (Positive/Negative)? Why do you view it this way?
- What kind of experiences have you faced while providing information about the cause of death?
- Do you consider the collection of COD data effective? If yes, why? If no, why not? Please explain in detail.
- What challenges do you think arise while collecting such data? Why do you think these challenges exist?
- Would you like to share any specific experiences? If so, please provide details.
- What is your opinion on the usage of this data? How effective do you think this data can be for practical use?
- To what extent do you believe COD data collection from burial sites can help in accurately identifying causes of death? If effective, why? If not, why not?

Opinions

- Can you suggest any better methods for collecting COD data from burial sites?
- What do you think are the advantages and disadvantages of collecting COD data from burial sites? Please explain in detail.

Qualitative Guideline for Conducting In-depth Interview with Verbal Autopsy Participants

General Information

- Age, marital status, education, occupation, etc. of the respondent.

Verbal Autopsy-Related Information

- How do you perceive the importance of cause-of-death (COD) data from burial sites? (Positive/Negative)? Why do you view it this way?
- Have you ever provided information about the cause of death of a deceased individual? If so, what kind of experiences did you face during the process?
- Would you like to share any specific experiences? If so, please provide details.
- Do you consider the collection of COD data effective? If yes, why? If no, why not? Please explain in detail.
- What challenges do you think arise while collecting such data? Why do you think these challenges exist?
- What is your opinion on the usage of this data? How effectively do you think it can be utilized?
- To what extent do you believe COD data collection from burial sites can help in accurately identifying causes of death? If effective, why? If not, why not?

Opinions

- Can you suggest any better methods for collecting COD data from burial sites?
- What do you think are the advantages and disadvantages of collecting COD data from burial sites? Please explain in detail.
